# Practical Points

**Published:** 1894-04-14

**Authors:** 


### PRACTICAL POINTS.

[Questions are invited for this column on any point in the administration of hospitals and asylums which may prove of difficulty or interest to our readers. All communications should be marked "Practical Points," addressed to the Editor of The Hospital, 428, Strand, W.C.]

*Hospital Construction.—Ward Floors.—"A Hospital Matron" writes to us as follows: I am much in want of information on a point of hospital construction. What is the best kind of floor suitable to a hospital of about 40 beds, where the old floors are much in need of renewal? The floors must be of a kind easily applied to a building badly built over 60 years ago, and so relatively cheap as to be within the means of an institution which is far from rich.*

The ward floors ought to be relaid with either oak or teak, in three inch widths, and side and secret nailed and tongued. When laid, the boards should either be paraffined or wax polished, and thereafter dry rubbed. Such floors will last at least twice as long as those which have not been treated in this way, and so have to be continually washed. In the long run hard wood will be found cheaper than deal.

*Hospital Wards.—"A Hospital Secretary" writes: Would you kindly give me your opinion as to (1) treating medical and surgical cases in the same ward? (2) Is it advisable to have one ward over another?*

(1) In the case of large hospitals it is not customary to treat medical and surgical cases in the same ward, although it is sometimes done. In smaller hospitals it is a common practice. Generally the medical opinion would in this case seem to be favourable rather than opposed to this system. (2) From the highest hygienic standpoint it is distinctly undesirable to have one ward over another, though such an arrangement is necessary in crowded cities where sites are difficult to secure. The ideal ward is one of a single storey built upon arches, through which the air can freely circulate so as to secure the maximum of ventilation and purity. In hospitals of more than one storey, attempts have been made with varying success to make each floor self-contained and distinct, and so secure the desired result.

